# Characterization of the Holliday Junction Resolving Enzyme Encoded by the *Bacillus subtilis* Bacteriophage SPP1

**DOI:** 10.1371/journal.pone.0048440

**Published:** 2012-10-31

**Authors:** Lisa Zecchi, Ambra Lo Piano, Yuki Suzuki, Cristina Cañas, Kunio Takeyasu, Silvia Ayora

**Affiliations:** 1 Departamento de Biotecnología Microbiana, Centro Nacional de Biotecnología, CSIC, Madrid, Spain; 2 Graduate School of Biostudies, Kyoto University, Sakyo-ku, Kyoto, Japan; Centro de Biología Molecular Severo Ochoa (CSIC-UAM), Spain

## Abstract

Recombination-dependent DNA replication, which is a central component of viral replication restart, is poorly understood in Firmicutes bacteriophages. Phage SPP1 initiates unidirectional theta DNA replication from a discrete replication origin (*ori*L), and when replication progresses, the fork might stall by the binding of the origin binding protein G*38*P to the late replication origin (*ori*R*).* Replication restart is dependent on viral recombination proteins to synthesize a linear head-to-tail concatemer, which is the substrate for viral DNA packaging. To identify new functions involved in this process, uncharacterized genes from phage SPP1 were analyzed. Immediately after infection, SPP1 transcribes a number of genes involved in recombination and replication from *P*_E2_ and *P*_E3_ promoters. Resequencing the region corresponding to the last two hypothetical genes transcribed from the *P*_E2_ operon (genes *44* and *45*) showed that they are in fact a single gene, re-annotated here as gene *44*, that encodes a single polypeptide, named gene *44* product (G*44*P, 27.5 kDa). G*44*P shares a low but significant degree of identity in its C-terminal region with virus-encoded RusA-like resolvases. The data presented here demonstrate that G*44*P, which is a dimer in solution, binds with high affinity but without sequence specificity to several double-stranded DNA recombination intermediates. G*44*P preferentially cleaves Holliday junctions, but also, with lower efficiency, replicated D-loops. It also partially complemented the loss of RecU resolvase activity in *B. subtilis* cells. These *in vitro* and *in vivo* data suggest a role for G*44*P in replication restart during the transition to concatemeric viral replication.

## Introduction

*Bacillus subtilis* SPP1 is one of the most intensively studied virulent phages from the Firmicutes phylum. It was described for the first time in 1968 by Riva and coworkers [1]. Phage SPP1 uses a headful packaging mechanism, so that ∼104% of the genome is packaged into the empty procapsids [2]. After injection into *B. subtilis* cells, the viral DNA, which is terminally redundant, circularizes by an unknown mechanism.

One of the most interesting properties of this phage is its replication process. The phage possesses two origins of replication, *ori*L and *ori*R, which are 12 -kb apart [3]. Replication starts according to the circle-to-circle mode (theta replication) from *ori*L, but it switches to a recombination-dependent replication (RDR) mode (sigma replication). It is believed that the origin binding protein, G*38*P, bound to *ori*R acts as a block in replication fork progression, and that this triggers the switch to late sigma replication [2]. The sigma replication mode is required to produce linear head-to-tail concatemers, which are the substrate for the viral packaging machinery [2]. The viral replication proteins are encoded by two early operons transcribed from promoters 2 (*P*_E2_) and 3 (*P*_E3_). The first operon, which is under the control of *P*_E2_, encodes essential enzymes required for theta replication [4], namely the origin binding protein, G*38*P [3], the helicase loader, G*39*P [5], [6], and the replicative helicase, G*40*P [7], [8]. Downstream of gene *40*, there are a set of hypothetical genes (*41* to *45*) that seem to be dispensable under laboratory conditions [2]. The second operon, which is under the control of *P*_E3_, encodes the essential single-stranded DNA binding protein (SSB), G*36*P, and essential enzymes required for the RDR mode, including the recombinase G*35*P [9], [10], and the 5′ → 3′ exonuclease G*34*.*1*P [11]. Additional host-encoded replication proteins are recruited by protein-protein interactions like those observed between the viral helicase and the host primase [12] or the τ subunit of the host DNA polymerase [13].

**Figure 1:**
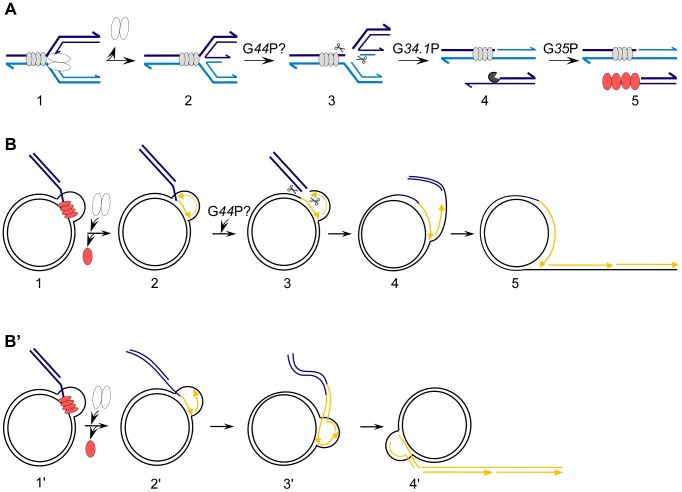
Putative roles of G*44*P in the generation of concatemeric linear SPP1 DNA after replication fork stalling. (A) The generation of a 3′-tailed dsDNA molecule. 1, G*38*P (grey ovals) bound to *ori*R may hinder the progression of the replisome (white ovals), causing its subsequent disassembly. 2, The fork reverses and a Holliday junction (HJ) is formed. 3, The G*44*P HJ resolvase (scissors) may cleave this substrate and a one-ended double strand break is formed. 4, The G*34*.*1*P 5′-3′ exonuclease (pacman) processes the end. 5, The G*35*P recombinase (red ovals) forms filaments on the generated 3′-tailed duplex DNA. The 3′-ends of the strands are shown by arrows. From this substrate two alternatives were proposed to generate SPP1concatemeric DNA (B and B’). (B) Generation of concatemeric DNA by a sigma-like mechanism. 1, G*35*P promotes strand invasion on a supercoiled SPP1 molecule. 2, The replisome is recruited and the invaded strand primes DNA synthesis with subsequent dislodging of G*35*P. 3, G*44*P may cleave the strands of the replicated D-loop. 4 and 5, DNA synthesis followed by DNA ligation would generate the proper substrate for concatemeric DNA synthesis by a semiconservative mechanism. (B’) Bubble migration model for the generation of concatemeric DNA. 1′ and 2′, These steps are common in both avenues. 3′ and 4′, The replication bubble migrates and the newly synthesized strands are extruded, so that by this mechanism a concatemer is formed by conservative DNA synthesis, without an obvious need for a D-loop resolvase. In B and B’ the template DNA is drawn in black and newly synthesized DNA in yellow. The steps where G*44*P could participate are indicated by the scissors.

The current model of how SPP1 replication switches from theta to sigma replication involves a double strand break (DSB) and replication restart by homology-directed recombination. The proposed model has G*38*P licensing *ori*L, which lies within its own coding sequence, to initiate theta DNA replication. Some time later, replication fork stalling may occur due to the barrier formed by G*38*P tightly bound to the second origin, *ori*R (Figure 1A). This is followed by replication restart [14], [15], during which fork reversal anneals the nascent leading- and lagging-strand ends to create a Holliday junction (HJ) [16]. Resolution of the HJ by an uncharacterized enzyme leads to a one-ended DSB (i.e., a DNA *end* from *one* molecule, see [17]), which is resected by the G*34*.*1*P exonuclease to produce a 3′-ssDNA tailed duplex [11]. The G*35*P recombinase forms filaments on this intermediate and promotes DNA strand invasion on another supercoiled DNA phage molecule, leading to the formation of a displacement loop (D-loop) intermediate, as has been documented *in vitro* [9]. At this D-loop, a new replisome is loaded by the interaction of G*35*P with G*36*P (viral SSB) and G*40*P (viral replicative helicase) [9], [11]. After recruitment of the SPP1-encoded and host-encoded replication proteins, the invading strand primes DNA synthesis at the leading strand, and lagging strand synthesis starts by the action of DnaG primase.

Two alternative models of replication have been proposed to explain how a concatemer is formed from the D-loop. In the first model (Figure 1B), an uncharacterized D-loop-specific endonuclease processes this recombination intermediate [11]. Then, in a way similar to plasmid rolling circle replication, concatemeric DNA replication could start from the generated 3′-OH end [18]. In the second model (Figure 1B’), which does not require a D-loop-specific endonuclease, the 3′-tail of the invading molecule primes DNA synthesis and then the D-loop migrates (bubble migration), leading to conservative concatemeric DNA synthesis [19].

**Figure 2:**
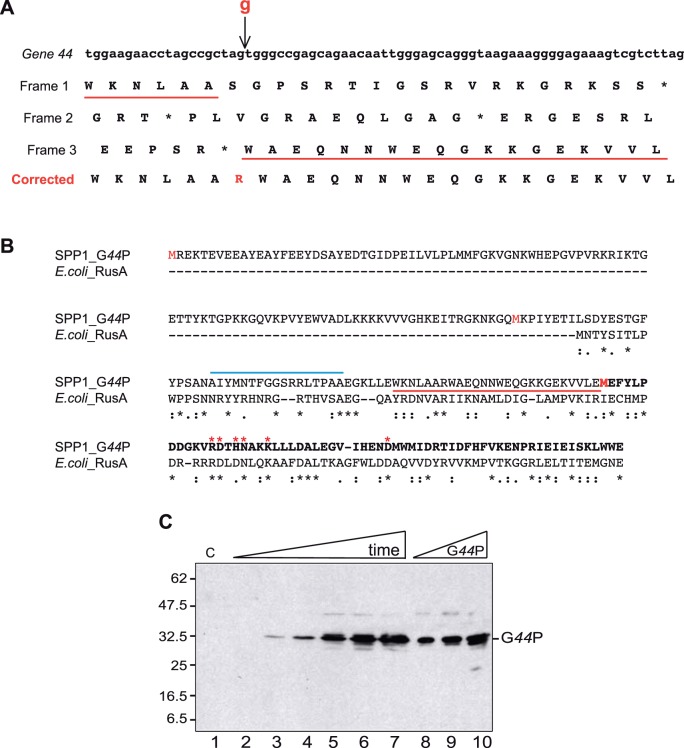
Identification of the correct G*44*P protein. (A) Part of the gene *44* carrying the sequence error. The arrow indicates the position of the missing guanine. Under the nucleotide sequence, the three possible reading frames are listed. The change of frame caused by the insertion of the guanine is underlined in red. The corrected sequence is shown in the bottom row. (B) Alignment with the ClustalW2 program of *Eco*RusA with the corrected sequence of G*44*P. In the G*44*P sequence the residues corresponding to former G*45*P are shown in bold. The region highlighted in part A is underlined in red, and the three putative starting methinonine residues are highlighted in red. The *Eco*RusA residues essential for catalysis are highlighted by red stars, and the region suggested to be involved in DNA binding and sequence specific cleavage is highlighted by a blue line. (C) G*44*P accumulation in the cell after SPP1 infection. *B. subtilis* cells were infected at an m.o.i of 10 and aliquots were taken every 5 min (from 0 [lane 2] to 25 [lane 7] min after infection). Crude extracts were prepared. Proteins (30 µg total protein) were separated in 15% SDS-PAGE and immunoblotted. As a control, 10, 20 and 40 ng of purified G*44*P were loaded (lanes 8-10). G*44*P was detected in the blots using anti-G*44*P rat polyclonal antisera. C: Crude extracts of non-infected cells. The running positions of the molecular weight markers are shown on the left.

Hence, a central step in the current model of replication switching is the activity of an unknown D-loop and HJ resolvase, which would cleave these three-stranded (D-loop) and four-stranded (HJ) recombination intermediates [15]. In the search for this specific endonuclease, we turned our attention to the small G*45*P protein (7.5 kDa, accession number Q38073), whose gene is under the control of the early *P*_E2_ promoter. G*45*P is distantly related to the RusA protein, which is encoded by the defective *Escherichia coli* Rac prophage (called *Eco*RusA in this work [20]). *Eco*RusA is a genuine HJ resolvase [21], [22], [23]. However, in contrast to *Eco*RusA and other members of the RusA superfamily (Pfam number 05866), which have an average size of 120 amino acids, the deduced amino acid sequence from the putative G*45*P protein revealed a 59-residue polypeptide [24]. The putative protein lacked the N-terminal region of *Eco*RusA, where residues important for binding to DNA and for specificity of cleavage are located [23] (see Figure 2). We show here that there was a sequencing error in the previously published SPP1 sequence, and that the correct nucleotide sequence consists of a single open reading frame (ORF) that encodes a 236-residue polypeptide (27.5 kDa), which is only 2 residues longer than the combined sequence of the segments formerly known as G*44*P and G*45*P. We have renamed this entire region G*44*P. We show here that full length G*44*P is produced *in vivo* and might be involved in DNA replication and/or repair processes. We describe how G*44*P binds specifically to a variety of DNA replication and recombination intermediates, including HJs and D-loops. From these substrates HJs and replicated D-loops are cleaved. In the light of these findings, the role of this resolving enzyme on SPP1 RDR is discussed.

## Materials and Methods

### Bacterial Strains, Plasmids and Phages

*E. coli* XL1 Blue and BL21 (DE3) pLysS were used for cloning and protein overexpression, respectively. To overexpress G*44*P, SPP1-encoded gene *44* was PCR amplified and cloned into *Nco*I-*BamH*I-cleaved pET-15b plasmid to generate plasmid pET-G*44*P. The sequence of gene *44* in plasmid pET-G*44*P was confirmed by nucleotide sequence analysis. *E. coli* BL21 (DE3) pLysS cells were transformed with pET-G*44*P plasmid and overexpression was induced by adding IPTG.

To assess whether G*44*P is able to complement a Δ*recU* mutant, gene *44* was PCR amplified and cloned into an *Xba*I-*Sph*I-cleaved pDG148 *B. subtilis* vector [25]. Several deletions and mutants were obtained, which suggested the toxicity of the gene, as was also observed in early attempts to clone this region in λ phages [26]. From the few transformants obtained, one containing plasmid pDG*44*P-M210V, which carries a conserved substitution at amino acid 210, was selected for *in vivo* analysis. The *B. subtilis* strain BG214 was used for phage amplification [27]. The strain TF8A has been previously described [28]. The SPP1 phages (wild-type [wt] and ΔA phages [24]) were amplified in BG214 cells grown in Luria-Bertani (LB) medium supplemented with 10 mM MgCl_2_.

### Survival Assays

Exponentially growing Δ*recU* cells (BG855; [29]) bearing plasmid pDG148 or pDG*44*P-M210V were obtained by inoculating overnight cultures in fresh LB media and growing to an OD_560nm_ of 0.4 at 37°C. The cultures were then exposed to 10 mM methyl methanesulfonate, and the fraction surviving at different times was determined by plating appropriate dilutions on LB plates.

### *In vivo* G*44*P Detection

BG214 cells were grown up to exponential phase (OD_560nm_  = 0.4) and then phage SPP1 was added with a multiplicity of infection (m.o.i.) of 10. One-ml samples were collected every 5 min, centrifuged, and stored at −20°C. Each pellet was resupended in 100 µl lysis buffer (50 mM Tris-HCl pH 7.5, 1 mM EDTA, 0.8% SDS, 150 mM NaCl, 1 mg/ml lysozyme). The mixtures were incubated for 30 min at 37°C and after sonication, 50 µl were subjected to 12.5% SDS-polyacrylamide gel electrophoresis (SDS-PAGE). For western blotting, the gel samples were transferred onto PVDF membranes and probed with rat polyclonal antibodies raised against G*44*P.

### Analysis of SPP1 Replication by Pulsed Field Gel Electrophoresis (PFGE)

*B. subtilis* cells were grown in LB containing 10 mM MgCl_2_ till OD_560 nm_  = 0.4 and infected with SPP1 or their mutants at an m.o.i of 5. At given times, 1 ml of culture was removed, rapidly placed on a water-ice mixture and centrifuged for 1 min at 14,000 rpm. The pellets were frozen and stored at −80°C. Samples were resuspended in 200 µl of buffer P1 (Qiagen) containing RNAse (0.1 mg/ml) and lysozyme (0.5 mg/ml), and lysed for 30 min at 30°C. Deproteinization was performed with Proteinase K (0.5 mg/ml) and SDS (0.8%) for 30 min at 30°C. 20 µl were directly loaded on a 0.8% agarose gel. PFGE was performed on a Bio-Rad CHEF-DR II apparatus. Running conditions were 4.5 V/cm, 0.5% TBE, 0.5–12 switch time for 17 h at 14°C. The molecular weight marker used was LW range PFG marker from New England Biolabs. The probe used for Southern blot development was a PCR product of 500 bp complementary to the gene *35* region. Southern blots were performed with Hybond-N+ membranes as instructed by the manufacturer (GE Healthcare) and detection was done with the AlkPhos Direct Labeling kit (GE Healthcare).

### Protein Purification and Molecular Mass Determination

G*44*P was overproduced in *E. coli* BL21 (DE3) pLysS cells containing plasmid pET-G*44*P. Expression of the protein was obtained by addition of 1 mM IPTG to the cells growing at 37°C. Cells were harvested and resuspended in buffer A [50 mM phosphate (pH 7.5), 5% glycerol, 1 mM EDTA] containing 200 mM NaCl, and lysed by sonication. Most of the protein was found in the pellet and recovered from it by washing at 500 mM NaCl. The resulting fraction was precipitated at 45% ammonium sulfate saturation, and loaded on a hydroxyapatite (Bio-Rad) column at 8 mM phosphate. The hydroxyapatite column was washed stepwise with increasing phosphate concentrations in buffer A containing 1 M NaCl, and the fractions containing G*44*P (30–60 mM phosphate) were dialyzed to a final NaCl concentration of 250 mM. The protein was then loaded on a MonoQ column (GE Healthcare) and finally eluted at 400 mM NaCl. The purity of the protein was assessed by 15% SDS-PAGE.

FPLC-gel filtration chromatography on a Superdex 75 column was carried out in buffer A containing 1 M NaCl with a flow rate of 0.5 ml/min, and the A_280_ was measured. 30 µg of protein in a volume of 200 µl were applied. A standard curve of K_av_ versus log_10_ of molecular mass was determined. Protein standards used were: RNase (13 kDa), chymotripsinogen A (25 kDa), ovalbumin (44 kDa), and albumin (67 kDa). The G*44*P concentration was determined using a molar extinction coefficient (at 280 nm) of 60,400 M^−1^ cm^−1^, and is expressed in the *Results* as the concentration of protein dimers.

### DNA Binding and Cleavage Reactions

For construction of the different DNA structures, combinations of oligonucleotides were used (see Table S1 and Figure S1). Oligonucleotides were labeled at the 5′-end by polynucleotide kinase with [γ-^32^P]-ATP. Annealing was performed in 100 mM phosphate buffer (pH 7.5) with the appropriate combinations of oligonucleotides, mixing one radiolabeled oligonucleotide with cold complementary oligonucleotides in a 1∶2 ratio. The annealed products were resolved on a 10% non-denaturing polyacrylamide gel. The bands containing the annealed substrates were excised and DNA was eluted into buffer containing 0.5 M ammonium acetate, 0.1% SDS and 1 mM EDTA, followed by incubation overnight at 4°C and ethanol precipitation. DNA concentration was calculated by scintillation counting. Binding of G*44*P to DNA was analyzed through electrophoretic mobility-shift assays (EMSA), using different radiolabeled DNA substrates (0.2 nM). Reactions were performed in buffer B (50 mM Tris-HCl pH 7.5, 50 ng/µl BSA, 50 mM NaCl, 5% glycerol) with 1 mM EDTA for 15 min at 37°C. Complexes were separated by 6% PAGE, and the gels were dried before autoradiography.

Cleavage of the DNA substrates (0.2 nM) by G*44*P was assayed at 37°C for 30 min in buffer B containing 10 mM MgCl_2_. Reactions (10 µl) were stopped by addition of 25 mM EDTA and heating at 95°C for 10 min. Reaction products were analyzed by 20% denaturing PAGE. A G+A sequencing ladder of the corresponding oligonucleotide was generated as described [30] and loaded on the gels as a marker.

### Atomic Force Microscopy (AFM)

The χ-structure is a plasmid-based HJ that was obtained as described previously [31]. In short, it is produced *in vivo* using the *E. coli* strain RM40, which carries an IPTG inducible *xerC* gene and harbors plasmid pSD115 with two *cer* sites [32]. The culture was grown at 37°C until mid-log phase and induced with 2 mM IPTG. After 90 min of incubation, cells were harvested and the plasmid-containing HJ intermediates were purified by standard alkaline lysis. The *Hin*cII-cleaved χ-structures were gel purified. The χ-structure has four arms of 620, 773, 1,565, and 1,990 bp, calculated assuming that the crossover occurs in the middle of the *cer* site. To visualize the binding of G*44*P to χ-structures by AFM, reaction mixtures were assembled in a tube before deposition onto a mica surface. The reactions contained 10 nM of G*44*P, 0.4 ng/µl plasmid-based HJ DNA, 50 mM Tris-HCl (pH 7.5) and 1 mM EDTA in a total volume of 10 µl. After incubation at 37°C for 20 min, the mixture was dropped onto a freshly cleaved mica surface, which had been pretreated with 10 mM spermidine. After 5 min incubation at room temperature, the mica was rinsed with water and dried under nitrogen gas. All imaging was performed in air using the cantilever tapping mode. The cantilever (OMCL-AC160TS-W2, Olympus) was 129 µm in length with a spring constant of 33–62 N/m. The scanning frequency was 1–2 Hz, and images were captured using the height mode in a 512×512 pixel format. The obtained images were plane-fitted and flattened by the computer program supplied by the imaging module before analysis.

## Results

### The SPP1 Genome Encodes a Protein Distantly Related to *Eco*RusA

With the aim of identifying SPP1-encoded proteins that may act at fork collapse and replication restart, and taking into account the apparent lack in G*45*P of some important residues of a HJ resolving enzyme [23], the region of the early *P*_E2_ operon that includes the genes *44* and *45* was re-sequenced. As shown in Figure 2A, we observed an additional G residue between positions 40238 (A) and 40239 (G) of the SPP1 sequence, leading to a shift of the frame. The hypothetical genes *44* and *45* became a single ORF, coding for a fused polypeptide of 236 residues. The C-terminal region of this 27.5 kDa protein, which we called G*44*P, shares homology with *Eco*RusA (Figure 2B). The RusA family of HJ resolving enzymes is widely distributed in phages and bacteria, with more than 700 representatives (Pfam 05866). With almost no exceptions, the proteins are approximately 120 residues long, which is in high contrast with the predicted length of G*44*P. Alternatively, translation might start from an internal methionine at codon 104 (Figure 2B), producing a polypeptide of 15.6 kDa (133 residues). This alternative construct is closer in size to RusA-like enzymes and has an overall identity to *Eco*RusA of 22.5% using the ClustalW2 program.

To determine which construct was produced *in vivo*, *B. subtilis* cells were infected with phage SPP1, and aliquots were taken at several times after infection and analyzed by western blot with polyclonal antibodies raised against purified G*44*P (see below). The synthesis of the 27.5 kDa protein (with a mobility of ∼32.5 kDa) was confirmed, but a polypeptide with a mass of ∼15.6 kDa was not detected (Figure 2C). The protein recognized by the anti-G*44*P polyclonal antibodies was detected 5 min after infection, and it was synthesized during the entire phage life cycle. Previously it was shown that transcription from the *P*_E2_ promoter starts 2 min after infection, and that the bulk of early transcription is complete by about 14 min, which is coincidental with the initiation of the late transcription 12 min after infection [2]. However, the replication genes are transcribed from *P*_E2_ and *P*_E3_ during the entire phage life cycle, suggesting a complex tuning of the SPP1 transcriptional program [4], [33]. Furthermore, from the synthesis kinetics we could not rule out a potential involvement of G*44*P in viral DNA packaging, as has been shown for the HJ resolvase encoded by the T4 bacteriophage [34].

### An SPP1 Variant, SPPlΔA, Shows a Delay in DNA Synthesis

Gene *44* is located within the large *Eco*RI fragment 1 of the SPPl genome [35]. Previously, viable SPPl deletion mutants mapped to this region were isolated [36]. One of these, SPPlΔL, carried a large deletion: 4.6-kb, encompassing the early *P*_E1_ operon (genes *46* to *53*) and six genes from the *P*_E2_ operon (genes *42* to *45*). SPPlΔL exhibited poor phage yield and small-plaque formation [24]. It was suggested that the poor growth phenotype associated with this deletion mutant could be due to premature transcriptional read-through from the *P*_E2_ early promoter onto the late genes coding for the terminase (genes *1* and *2*), the portal protein (gene *6*) and the accessory protein (gene *7*), resulting in defects in packaging [24]. However, another possibility could be that G*44*P, which is missing in SPPlΔL, is required for phage amplification, or for the processing of concatemers, so that the absence of gene *44* is responsible for the observed phenotype.

To discriminate between the two possibilities, and to analyze the role of G*44*P in the SPP1 replication cycle, we focused on another deletion mutant, SPPlΔA, which carries a shorter deletion (∼3 kb) than SPPlΔL [35], and showed a less pleiotropic phenotype. The exact extent of the deletion carried by SPPlΔA was unknown. Therefore, we first mapped the SPPlΔA deletion by sequencing the deletion endjoints with appropriate primers. The sequence analysis showed that a segment of DNA between two short, directly repeated sequences of 6 bp (AGCGGC) located 3120 bp apart in the SPP1 sequence was absent. This deletion encompasses genes *43* to *44* from the *P*_E2_ operon and genes *46* to *51* from the *P*_E1_ operon. It is expected that this deletion mutant would not show premature transcription of the late genes, because the genuine terminators of the early *P*_E1_ operon are present, and that any phenotype observed should be due to the lack of an early expressed gene. *In vivo* the SPPlΔA mutant exhibited small-plaque formation, whereas phage yield after 2 h of infection was similar to levels obtained with the wt phage.

PFGE experiments were used to analyze the rate of synthesis and the structure of viral DNA that accumulated in wt SPP1 and in SPPlΔA infections. Previously, it was shown that SPP1 theta-type replication initiates at min 3 after infection, RDR initiates at min 7, and processive packaging of a concatemer formed by ∼4–5 genome equivalents into empty viral procapsids initiates at min 12 post-infection [3], [4], [33], [37]. In wt SPP1-infected cells the accumulation of discrete DNA molecules with a size of 1–5 viral genomes was observed (Figure S2). As expected, an infection with the control mutant SPP1*sus19*, which carries a mutation in the gene coding for the large terminase subunit G*2*P [33], accumulated under non-permissive conditions only concatemeric DNA corresponding to 4–5 genome equivalents (data not shown). In SPPlΔA-infected cells, the accumulation of intermediates corresponding to 1–5 viral genomes was also observed, but DNA synthesis was 2- to 3-fold lower in comparison with wt SPP1-infected cells (Figure S2). This is the first observation of an SPP1 mutant with a DNA synthesis delay phenotype due to a delay in initiation or to slower DNA synthesis. SPPlΔA only lacks two early genes from the *P*_E2_ early replication operon (genes *43* and *44*). Since it was predicted that G*43*P is a transcriptional regulator of late expressed genes ([2] and unpublished results), it is possible that the observed phenotype is due to the absence of G*44*P.

These results suggest that the absence of G*44*P delays SPP1 replication, but that G*44*P is not essential for phage amplification. Similarly, the RecU HJ resolving enzyme is not essential [38]. We hypothesized that any of the redundant host-encoded resolving/dissolving enzymes could complement the absence of G*44*P in the SPP1ΔA mutant. Our wt strain, *B. subtilis* BG214, carries the SKIN prophage [27]. The SKIN-*yqaN* gene, which is under the control of *sknR* [39], codes for YqaN, a protein homologous to RusA (30% identity over a 50-amino acid stretch). In order to check if YqaN could complement the absence of G*44*P, the *B. subtilis* TF8A strain, which lacks the SKIN prophage [28], was infected with the SPP1ΔA mutant. No difference in phage yield was observed. Furthermore, when the *B. subtilis* BG855 strain, which lacks host-encoded RecU [29], was infected with SPP1ΔA, no difference in phage yield was observed relative to the *rec* ^+^ cells. It is likely that G*44*P is not required for phage amplification under laboratory conditions, or that a not yet identified function from *B. subtilis* can perform its activity.

**Figure 3:**
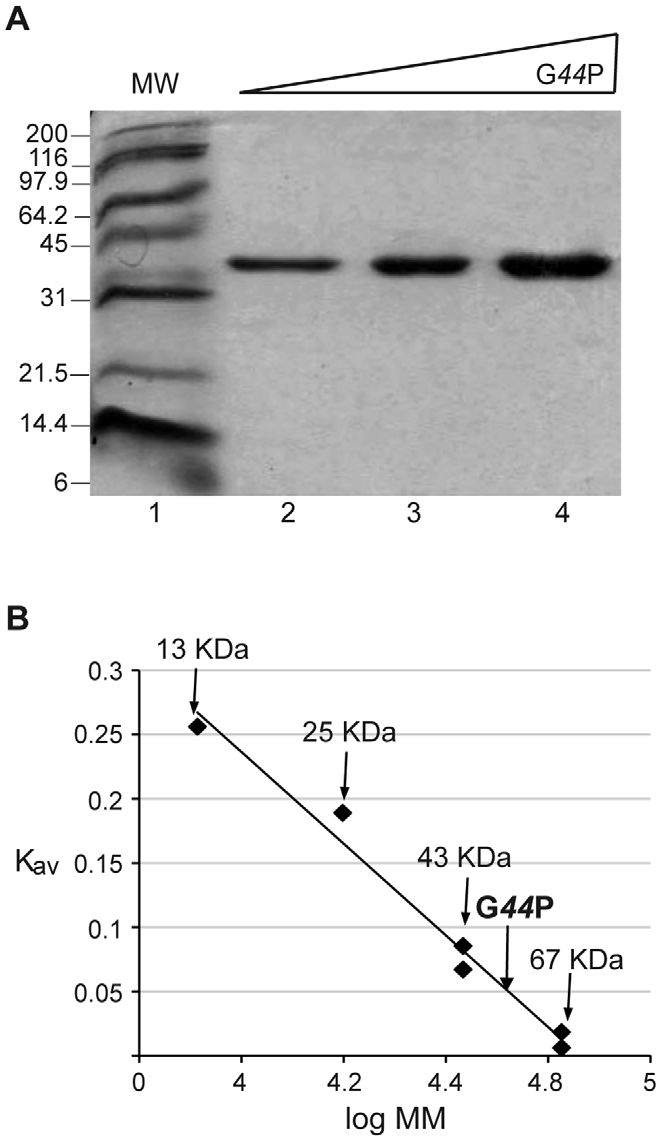
Purified G*44*P is a dimer in solution. (A) Electrophoresis of the purified protein on a 15% SDS-polyacrylamide gel. Lane 1: molecular weight marker; lanes 2-4: increasing concentrations of G*44*P (1–4 µg). (B) Estimation of the molecular mass of G*44*P by gel filtration chromatography on a Superdex 75 column. A standard curve of K_av_ versus log_10_ of molecular mass of protein standards was determined. The K_av_ of G*44*P was 0.05, corresponding to a molecular mass of 54 kDa.

### A G*44*P Variant Partially Complements Loss of RecU Activity

To test the hypothesis that G*44*P could be involved in DNA repair processes, BG214 (*rec* ^+^) competent cells were transformed with a plasmid-borne gene *44* construct. DNA from plasmid-borne gene *44* produced either no or few transformants (∼1000-fold lower transformation frequency than the control plasmid vector). Analysis of this low number of transformants revealed that the plasmids suffered structural rearrangements, such that in only a very few cases were plasmids carrying a full-length gene *44* obtained. Nucleotide sequence analyses revealed that all full-length clones contained mutation(s) within gene *44*. One of these mutations was a conserved substitution in gene *44* of a methionine at position 210 for valine (M210V); in an equivalent position *Eco*RusA has a valine. The Δ*recU* strain (BG855) was transformed with the plasmid-borne gene *44*-M210V variant, and cells were transiently exposed to 10 mM methyl methanesulfonate for varying times. As revealed in Figure S3, G*44*P-M210V partially complemented the DNA repair defect of Δ*recU* cells. We conclude that: i) gene *44* is toxic for *B. subtilis* cells, and ii) G*44*P-M210V partially replaces the activity of the RecU HJ resolving enzyme.

### G*44*P is a Dimer in Solution

Since the C-terminal half of G*44*P shares a significant degree of identity with DNA resolvases of the RusA family (Figure 2), and *in vivo* G*44*P-M210V partially complemented the *recU* defect (Figure S3), the interaction of G*44*P with DNA and its hypothetical role in DNA recombination was investigated *in vitro*. SPP1 gene *44* was PCR amplified and cloned into the expression vector pET-15b. After IPTG induction, we observed the appearance of a protein at ∼32 kDa, although the expected mobility was 27.5-kDa. G*44*P was purified, and its identity confirmed by peptide mass fingerprinting using the Mascot program. Purified G*44*P was more than 99% pure as judged by Coomassie Blue staining after SDS-PAGE (Figure 3A). The native state of soluble G*44*P (27.5 kDa) was evaluated using an FPLC-Superdex 75 gel filtration column. The protein eluted in a volume that corresponded to ∼54 kDa on the standard protein curve, which is consistent with it being a dimer in solution (Figure 3B).

**Figure 4:**
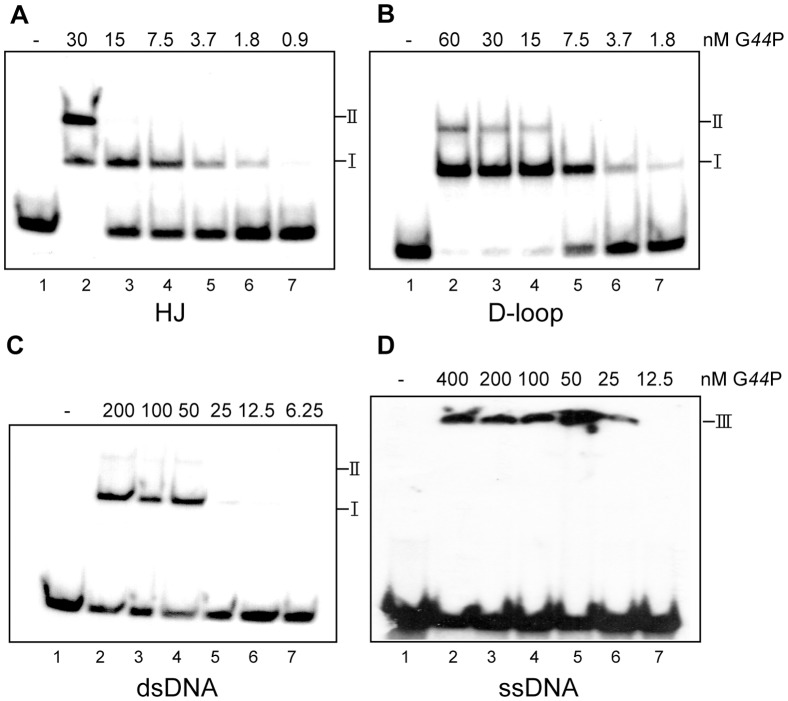
G*44*P binding to different DNA substrates. EMSAs showing binding of G*44*P to the indicated [γ^32^P]-labeled DNA substrates: (A) HJ-J3, (B) D-loop DL-D, (C) 80-bp dsDNA, and (D) 80-nt ssDNA. DNA (0.2 nM) was incubated with increasing amounts of G*44*P as indicated in buffer B containing 1 mM EDTA for 20 min at 37°C. The three types of complexes formed are denoted by I, II, and III.

### G*44*P Binds Recombination Intermediates

To learn whether G*44*P binds recombination intermediates, the binding to single-stranded (ss) DNA, double-stranded (ds) DNA, HJs and D-loops was analyzed. The different substrates constructed to test G*44*P binding are described in Figure S1 and Table S1. [γ-^32^P]-labeled DNA substrates were incubated with increasing G*44*P concentrations in buffer B containing 1 mM EDTA, and the complexes were visualized by EMSA followed by autoradiography. As revealed in Figure 4, G*44*P preferentially binds D-loops (DL-D) and HJs (HJ-J3). With D-loops and HJs, G*44*P exhibited some cooperativity in the binding to DNA, and formed two types of complexes (I and II, Figure 4A and 4B). From these assays we could calculate an apparent binding constant (K_app_, or the protein amount where 50% of the DNA is complexed with the protein) of ∼5 nM for D-loop DNA, and ∼10 nM for HJ DNA. G*44*P bound dsDNA with a K_app_ of ∼48 nM, and two complexes were observed (Figure 4C). However, in the EMSA, G*44*P-dsDNA complexes were unstable and often broken during electrophoresis (Figure 4C), and hence free DNA was observed even at saturating protein concentrations. G*44*P bound ssDNA with poor affinity and formed aggregates that did not enter into the gel (Figure 4D).

**Figure 5:**
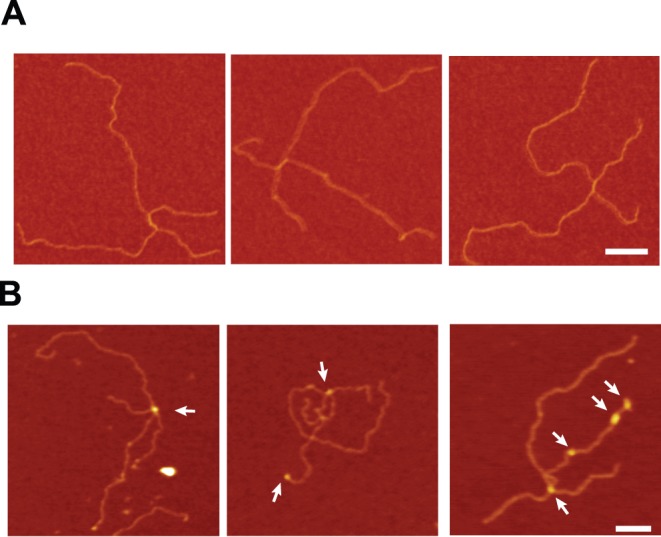
Visualization of G*44*P bound to HJ structures by AFM. (A) Representative AFM images of the χ-structure DNA alone and (B) after incubation with G*44*P (10 nM) in the presence of 1 mM EDTA. G*44*P was observed bound to the junction as well as to the dsDNA arms. Arrows indicate protein-DNA complexes. Scale bar  = 100 nm.

The interaction of G*44*P with HJ DNA was also analyzed by AFM. In this case, to visualize the HJ we used a longer plasmid-sized HJ, the χ-structure (with four arms of 620, 773, 1565 and 1990 bp; Figure 5A). G*44*P was incubated with χ-structures in the presence of 1 mM EDTA and complexes were visualized by AFM in the air mode. We observed proteins bound to the center of the junction, as well as proteins localized at sites other than the junction (Figure 5B). From the AFM results, it can be deduced that the two complexes observed by EMSA with the two recombination intermediates (HJs and D-loops) were a combination of high affinity binding to the junction (complex I) at low protein concentrations, followed by lower affinity binding at dsDNA regions (complex II) as the concentration of protein increases.

### G*44*P Resolves HJs with Relaxed Sequence Specificity

**Figure 6:**
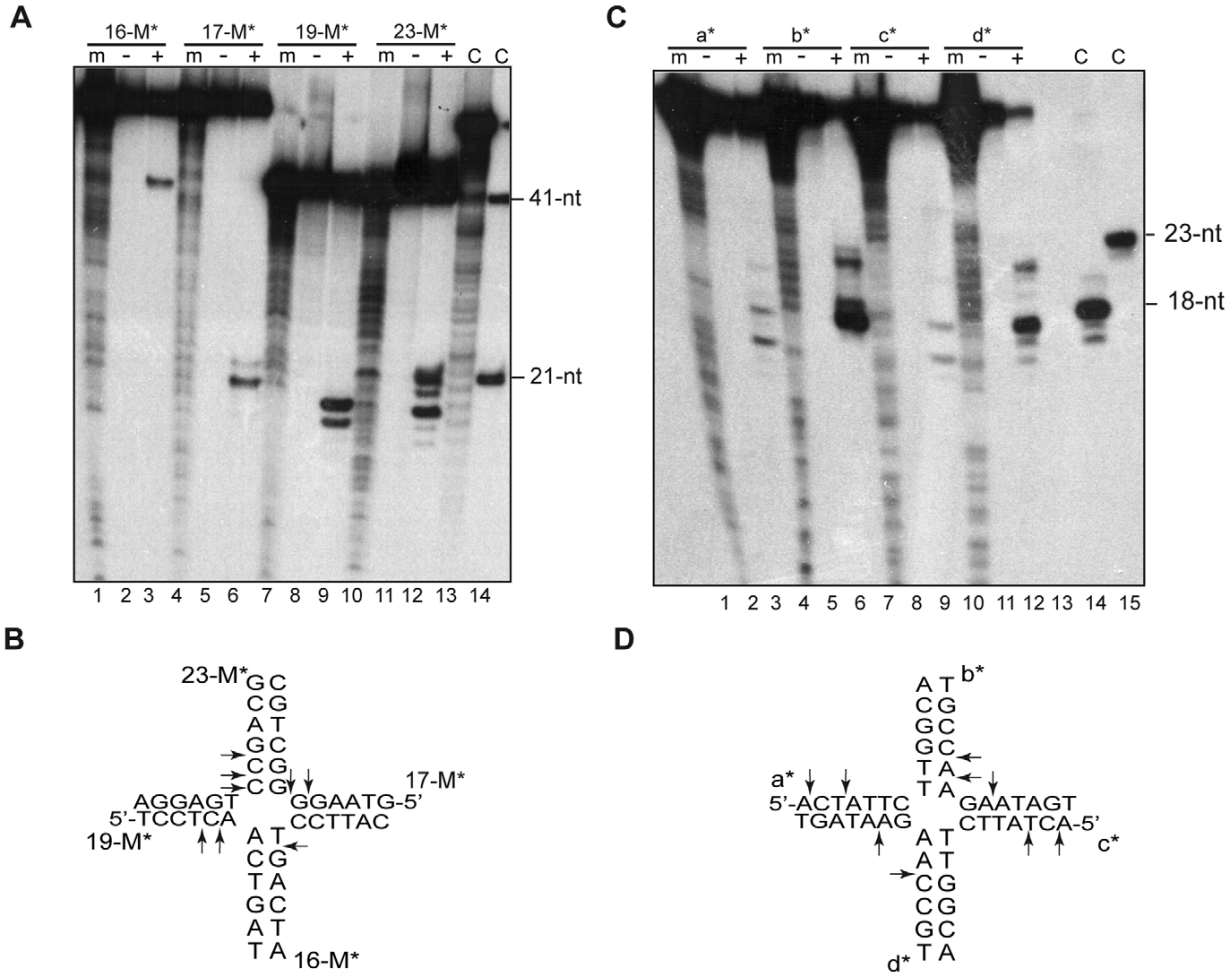
Determination of the cleavage ability of G*44*P on static and mobile HJs. (A and C) A fixed HJ (HJ-23M, in A) or a mobile HJ containing a 13-bp homologous core (HJ-jbm6, in C) [γ^32^P]-labeled at the indicated strand was incubated with 10 nM G*44*P in buffer B containing 10 mM MgCl_2_ for 30 min at 37°C. Reaction products were analyzed using 15% denaturing PAGE in the presence (+) or absence (-) of protein. “m” indicates the G+A sequencing ladder obtained for the corresponding labeled oligonucleotide. To serve as additional molecular weight markers, and denoted by C, 41-nt, 21-nt, 23-nt and 18-nt primers were loaded. In lane 13 of panel A, a degraded 17-M oligonucleotide was loaded. (B and D) The cleavage sites detected are indicated by arrows in the core of the two HJ sequences.

The current model of the shift in phage SPP1 from theta to concatemeric replication proposes that replication fork reversal generates a HJ intermediate (Figure 1A), and then a structure-specific endonuclease processes this intermediate, leading to a one-ended DSB, which is further processed by the G*34.1*P exonuclease so that a 3′-tailed duplex DNA is generated. This is the proper substrate for the strand invasion catalyzed by the G*35*P recombinase [11]. Hence, the activity of a HJ resolving enzyme seems crucial for SPP1 replication. As expected, G*44*P was unable to cleave HJs in the absence of Mg^2+^ ions (data not shown). We then analyzed whether G*44*P was able to resolve HJ intermediates in the presence of high Mg^2+^ concentrations (10 mM). G*44*P (10 nM) cleaved a fixed HJ structure (HJ-23M; see Table S1 and Figure S1 for detailed descriptions of the substrates) with symmetry, i.e., cleavage was observed in opposite arms, which accounted for the resolution of the structure (Figure 6A and 6B). The product of resolution could also be observed on native PAGE (data not shown). The sequence recognized in strand 17-M was 5′-AAG↓G↓GG-3′ and in strand 19-M 5′-CCT↓C↓AA-3′. Both cleavages occurred 1-2-bp from the junction, but no apparent consensus sequence was recognized at these cleavage sites. No endonuclease activity was observed with a dsDNA control that carried the 5′-CCTCAA-3′ sequence (data not shown). In addition, cleavages in strands 16-M and 23-M were also observed. In continued attempts to identify the consensus sequence of G*44*P cleavage, we analyzed the cleavage of a mobile HJ, HJ-Jbm6, which has a 13-bp homologous core. In theory, this junction can spontaneously branch migrate between this homologous core in order to locate the preferred sequences for resolution at the proper place [38]. The majority of the cleavage sites detected with this HJ were again at symmetric points along the b and d strands (Figure 6C and 6D). With this HJ, the preferred sites of cleavage were at the 5′-GCC↓AAG-3′ and 5′-AGA↓ATA-3′ sequences, but other sites were also cleaved, which suggests a limited sequence specificity.

### G*44*P Resolves Replicated D-loops

**Figure 7:**
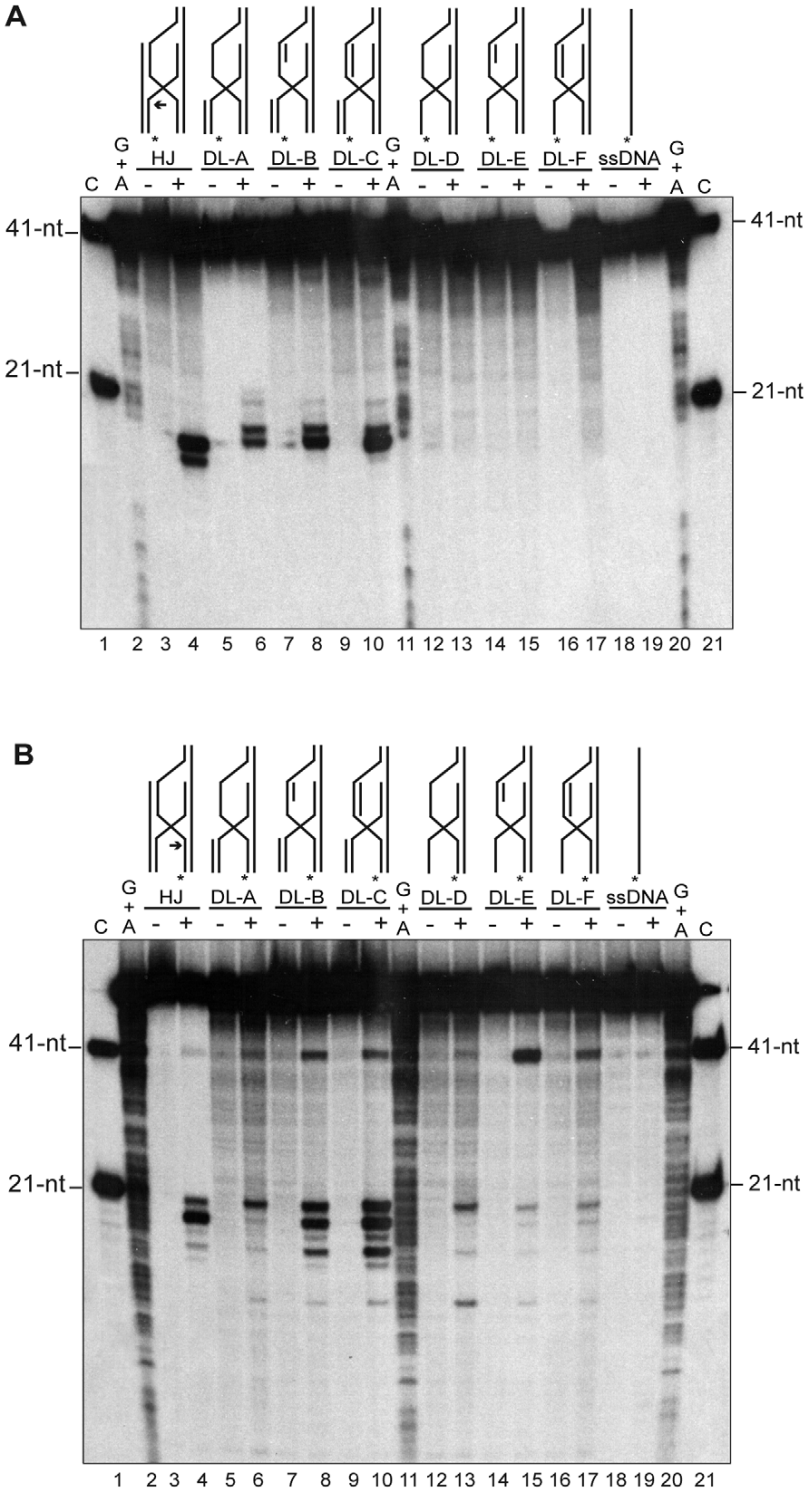
G*44*P-mediated cleavage of replicated D-loops. Different D-loop variants resembling several recombination intermediates were end-labeled at the 5′end of oligonucleotide 19-M (the invading strand, in A) or of oligonucleotide 17-M (the displaced strand, in B) and were incubated with 10 nM G*44*P in buffer B containing 10 mM MgCl_2_ for 30 min at 37°C. Reaction products were analyzed using 20% denaturing PAGE and revealed by autoradiography. Drawings indicate the different substrates analyzed (D-loops A to F, and control HJ and ssDNA). Asterisks indicate the [γ^32^P]-ATP labeling of oligonucleotides at the 5′end. In the HJ substrate, an arrow indicates the major cleavage site. As markers, the G+A sequencing ladder obtained for the corresponding labeled oligonucleotide and the 41-nt and 21-nt primers for the corresponding sequence were loaded.

In the previous section it was shown that G*44*P might generate the one-ended DSB by cleavage of the HJ formed after fork reversal, so that once this substrate is processed by the G*34*.*1*P exonuclease, the G*35*P recombinase will invade an intact viral copy to form a D-loop intermediate [11]. In one of the variants of the RDR model (Figure 1B) the involvement of a D-loop resolvase was proposed, which would generate a rolling circle-like intermediate. However, the model did not discriminate the level at which the putative D-loop resolving enzyme might work. This resolving enzyme could cleave the D-loop immediately after this intermediate has been formed, or replication could first restart from the D-loop, and then the intermediate (i.e., a replicated D-loop) could be cleaved by the resolvase. In order to test which of these recombination intermediates can be resolved by G*44*P, several substrates (based on HJ-23M, which is efficiently cleaved by G*44*P) were constructed by annealing the proper oligonucleotides (see Table S1 and Figure S1). These substrates would represent the control HJ-23M (Figure 7, lanes 3-4), synthetic versions of D-loops that mimic different recessed and/or replicated D-loops (DL-A to DL-F, Figure 7, lanes 5-17), and the ssDNA control (Figure 7, lanes 18-19). The invading strand (strand 19-M, Figure 7A) or the displaced strand (strand 17-M Figure 7B) was labeled in all the substrates and the cleavage was analyzed by denaturing PAGE after G*44*P incubation. As observed in Figure 7A, lanes 13, 15 and 17, G*44*P was unable to cleave a ssDNA invading a duplex. This oligonucleotide carries the 5′-CCTCAA-3′ sequence (see Table S1), but the sequence is located in the region of the oligonucleotide that remains single stranded after annealing, and therefore it was not recognized by G*44*P. Furthermore, the cleavage efficiency of the other D-loop variants (DL-A, DL-B and DL-C) depended not only on the dsDNA nature of this invading arm, but also on the dsDNA nature of the displaced strand. As shown in Figure 7A, the cleavage of the invading strand (strand 19-M) was more efficient when the displaced strand was a replicated D-loop (substrate DL-C, Figure 7A, lanes 9-10). When the cleavage of the displaced strand was analyzed, the results confirmed that the displaced strand had to be in the replicated form (i.e, in dsDNA form also in the bubble) to be cleaved by G*44*P, so that cleavage products were observed with the HJ substrate (Figure 7B, lanes 3-4) and with the replicated D-loops (DL-B and DL-C, lanes 7-10). In addition, a new cleavage product of 41-nt was observed in some of the substrates. These results indicated that G*44*P cleaves a regressed fork that has generated a HJ so that a one-ended DSB is generated, and that after that, it may participate in the processing of a recombination intermediate that resembles a HJ, which is formed after the reconstitution of an active replication fork from a D-loop.

## Discussion

When the complete DNA sequence of the SPP1 genome was published in 1997, 53 ORFs were predicted, and about half of them had an assigned function [40]. Among the unknown functions, there was one small ORF, coding for a 59-residue polypeptide (G*45*P) that was postulated to be a defective RusA resolvase [20]. After amendment of the sequence of the gene *44*–*45* interval (Figure 2), we have shown that SPP1 encodes a 27.5 kDa structure-specific endonuclease, which was not previously identified, and that the gene product preferentially cleaves branched DNA structures such as HJs and replicated D-loops. The *in vitro* results presented here would suggest an *in vivo* role for G*44*P in the shift from theta to sigma DNA replication, the type of replication required for the generation of the concatemeric DNA used for viral packaging. SPP1 mutants in the essential replication genes *38*, *39* and *40* show a D0 phenotype (absence of DNA synthesis), whereas mutants in replication genes *35* and 3*4.1* exhibit a DNA arrest (DA) phenotype [2]. However, SPP1ΔA, which lacks genes *43* and *44* from the early *P*_E2_ operon, shows a DNA delay (DD) phenotype, suggesting that G*44*P is not an essential protein, but that its absence delays viral amplification. The lack of essentiality of G*44*P contrasts with the essentiality of G*38*P, G*39*P, G*40*P (D0 phenotype) and G*34.1*P, G*35*P (DA phenotype) [41], [42]. In addition, no host-encoded function can replace G*38*P, G*39*P, G*40*P, G*34*.*1*P, G*35*P or G*36*P in theta and sigma viral DNA synthesis [15], but an unidentified host-encoded resolvase (or dissolvase) could complement missing G*44*P activity in our assays. In fact, *B. subtilis* is a naturally transformable bacterium, and the current model of how exogenous DNA integrates into the chromosome, if homology is present, suggests the activity in *B. subtilis* cells of an unknown endonuclease that will cleave a D-loop intermediate [43], [44]. This is consistent with the observations that: i) neither RecU nor YqaN catalyze such a resolution reaction *in vivo* [28], [43], and ii) D-loops are not cleaved *in vitro* by the RecU HJ resolvase, which specifically cleaves HJs [45].

Viral HJ or branch-specific endonucleases are ubiquitous in phages, and in the majority of the cases are non-essential proteins [15]. Currently, five viral resolvase superfamilies have been reported, represented by T4 endonuclease VII, T7 endonuclease I, λ Rap endonuclease, *Eco*RusA, and the phage encoded-RuvC-like enzymes [46]. Their *in vivo* roles in processes other than phage recombination suggest many different activities. Phage T4 endo VII (also known as gp49) is an essential enzyme that plays an important role in DNA encapsidation by de-branching RDR intermediates to make DNA substrates amenable to packaging. In fact, the resolvase is directly targeted to the packaging machinery by interactions with the gp20 portal protein [34], [47]. T7 endonuclease I (also called gene 3 endonuclease or gp3) is a non-essential enzyme involved in the cleavage of the host replication fork to produce DSBs, which can be further digested to produce nucleotide products used for phage DNA synthesis [48]. A similar role has been suggested for the *Lactococcus lactis* bIL66-encoded RuvC-like resolvase [49].

Lysogenic lambdoid phages and the lytic SPP1 have homologues of the *Eco*RusA HJ resolvase, or of the λ-encoded Rap structure-specific endonuclease [22], [46], and conservation of the gene arrangement between lambdoid phages suggests that Rap and RusA possess equivalent functions [50]. RusA homologs are present in numerous phages [22], [46]. The Rap function is active in the Red pathway of recombination (mediated by the non essential Redα-Redβ exonuclease/recombinase pair [51]), and other functions are unknown [52]. From the data presented in this work, a role for G*44*P in phage DNA recombination and repair can be hypothesized: (i) *in vivo,* a G*44*P point mutant can promote DNA repair, or at least partially complement a *B. subtilis* Δ*recU* deletion (Figure S3), (ii) G*44*P could participate in the resolution of the HJ formed after fork regression (Figure 6), and (iii) G*44*P could act in the resolution of the D-loop once replication has been established on this recombination intermediate (Figure 7). We cannot rule out, however, that G*44*P might also produce DSBs on host DNA, given the poor sequence specificity in the cleavage observed (Figure 6). Degraded DNA would be then used as a source of nucleotides for SPP1 DNA synthesis, as has been proposed for the T7 resolving enzyme [48].

RusA-like enzymes are the smallest of the HJ resolving enzymes described to date. *Eco*RusA is a small, Mg^2+^- and Mn^2+^-dependent enzyme composed of two identical 14-kDa subunits [21], [53]. Like *Eco*RusA, G*44*P is a dimer in solution, and cleaves recombination intermediates in the presence of Mg^2+^. *Eco*RusA binds not only to branched structures, but also to linear dsDNA and ssDNA. The affinity of *Eco*RusA for ssDNA is higher than for dsDNA [54]. In contrast, G*44*P binds very poorly to ssDNA (Figure 4), and its affinity for dsDNA is 5- to 10-fold lower than for the recombination intermediates.

The specificity of cleavage of RusA-like enzymes has been analyzed. *Eco*RusA seems to specifically cleave HJs, and almost exclusively 5′ to CC dinucleotides located symmetrically about the branch point [21], [23], [54]. In contrast, G*44*P from phage SPP1 cleaves both HJs and replicated D-loops (also known as nicked HJs) with a relaxed sequence specificity. Similarly, *r1t*RusA is less strict in its structure and sequence selectivity than the *Eco*RusA enzyme [22]. Unlike *Eco*RusA and *r1t*RusA, which cannot cleave static HJs [22], we observed with G*44*P efficient and symmetric cleavage of the two static junctions tested (HJ-23M and HJ-J3, Figure 6 and data not shown). How these differences are achieved remains to be determined. G*44*P contains an additional region of 13 kDa that is not present in the other RusA-like enzymes (Pfam 05866). The role of this extended N-terminal region remains unknown, but we speculate that it could contribute to some of the differences detected in this study.

## Supporting Information

Figure S1 **Construction of DNA** **structures used in this study.** DNA structures were made by annealing the proper oligonucleotides. The name of every oligonucleotide used to construct the structure is located at the 5′-end. In HJ-jbm6, the mobile core is represented by thicker lines.
(TIF)

Figure S2 **PFGE analysis of viral replication intermediates after wtSPP1 and SPP1ΔA infection.** *B. subtilis* cells were infected with the indicated phage at an m.o.i. of 5, and aliquots were removed at the given times and processed as described in Materials and Methods. DNA was resolved by PFGE in 1% agarose. The gel was (A) stained with ethidium bromide and (B) blotted to Nylon membranes and hybridized with a probe for SPP1. In A, the positions and the size of the markers are shown. In B, the different multimeric intermediates observed are highlighted.
(EPS)

Figure S3 ***In vivo*** **complementation of a** ***B. subtilis*** **Δ** ***recU*** **mutant.** Wt, Δ*recU* (pDG148) and Δ*recU* (pDG*44*P-M210V) strains were grown in LB medium at 37°C in the presence of 0.02 mM of IPTG to an A_560 nm_ of 0.4. The cultures were then exposed to 10 mM methyl methanesulphonate and the fraction surviving at 15 and 30 min was determined. The data are the means of three independent experiments.
(EPS)

Table S1 **Sequence of the oligonucleotides used for constructing DNA substrates.**
(DOC)
