# Supplementary figures and images for: Characterization of the Holliday Junction Resolving Enzyme Encoded by the Bacillus subtilis Bacteriophage SPP1
Source: PLoS One. 2012 Oct 31;7(10):e48440. doi: 10.1371/journal.pone.0048440 (PMC3485210; doi:10.1371/journal.pone.0048440)

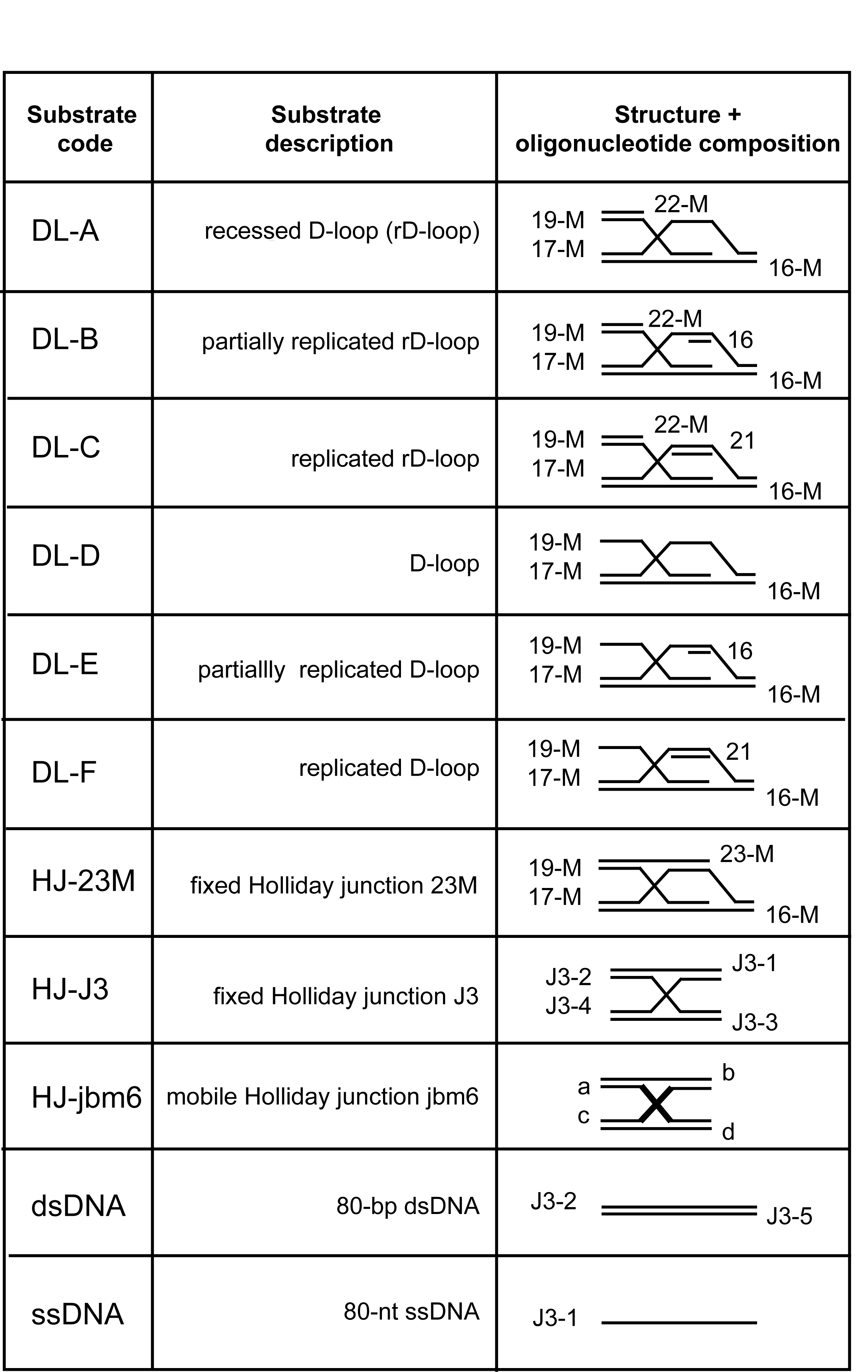

Supplement: Figure S1 — Construction of DNA structures used in this study. DNA structures were made by annealing the proper oligonucleotides. The name of every oligonucleotide used to construct the structure is located at the 5′-end. In HJ-jbm6, the mobile core is represented by thicker lines. (TIF) [file pone.0048440.s001.tif]
