# Supplementary material for: Characterization of the Holliday Junction Resolving Enzyme Encoded by the Bacillus subtilis Bacteriophage SPP1
Source: PLoS One. 2012 Oct 31;7(10):e48440. doi: 10.1371/journal.pone.0048440 (PMC3485210; doi:10.1371/journal.pone.0048440)
Supplement: Table S1 — Sequence of the oligonucleotides used for constructing DNA substrates. (DOC) [file pone.0048440.s004.doc]

Table 1. Sequences of the oligonucleotides used for constructing DNA substrates

| Name | Sequence |
| --- | --- |
| J3-1 | 5’-CGCAAGCGACAGGAACCTCGAGAAGCTTCCGGTAGCAGCCTGAGCGGTGGTTGAATTCCTCGAGGTTCCTGTCGCTTGCG-3’ |
| J3-2 | 5’-CGCAAGCGACAGGAACCTCGAGGAATTCAACCACCGCTCAACTCAACTGCAGTCTAGACTCGAGGTTCCTGTCGCTTGCG-3’ |
| J3-3 | 5’-CGCAAGCGACAGGAACCTCGAGTCTAGACTGCAGTTGAGTCCTTGCTAGGACGGATCCCTCGAGGTTCCTGTCGCTTGCG-3’ |
| J3-4 | 5´-CGCAAGCGACAGGAACCTCGAGGGATCCGTCCTAGCAAGGGGCTGCTACCGGAAGCTTCTCGAGGTTCCTGTCGCTTGCG-3’ |
| J3-5 | 5’-CGCAAGCGACAGGAACCTCGAGTCTAGACTGCAGTTGAGTTGAGCGGTGGTTGAATTCCTCGAGGTTCCTGTCGCTTGCG-3’ |
| 16-M | 5′-GACGCTGCCGAATTCTACCAGTGCCTTGCTAGGACATCAGTCCTTACCTGCAGGTTCAC-3′ |
| 17-M | 5′-GGGTGAACCTGCAGGTAAGGGGCTGCTCATCGTAGGTTAGTTGGTAGAATTCGGCAGC-3′ |
| 19-M | 5′-TAAGAGCAAGATGTTCCTCAACTGATGTCCTAGCAAGGCAC-3′ |
| 22-M | 5′-TGAGGAACATCTTGCTCTTA-3′ |
| 23-M | 5′-ACTAACCTACGATGAGCAGCCTGAGGAACATCTTGCTCTTA-3′ |
| 21 | 5’-ACTAACCTACGATGAGCAGCC-3´ |
| 16 | 5’-ACTAACCTACGATGAG-3´ |
| Jbm6-a | 5’-GCGTTACAATGGAAACTATTCTTGGCAGTTGCATCCAACG -3’ |
| Jbm6-b | 5´-CGTTGGATGCAACTGCCAAGAATAGTGTCAGTTCCAGACG-3´ |
| Jbm6-c | 5´-CGTCTGGAACTGACACTATTCTTGGCAAATGGTCGTAAGC-3´ |
| Jbm6-d | 5´-GCTTACGACCATTTGCCAAGAATAGTTTCCATTGTAACGC-3´ |
